# Nine Nevers

**Published:** 1859-10

**Authors:** 


### NINE NEVERS.

NEVER write a letter or a line in a passion.

Never spit or blow your nose on the sidewalk.

Never find fault until you are as sure as you are of your existence that a fault has been committed.

Never say what you would do under any given circumstances.

Never disparage another by name in a letter.

Never get in a rage.

Never utter a syllable in a passion.

Never refuse to pay a debt when you have the money in your pocket.

Never take physic until you have tried patience.

